# A Complete and Low-Cost Cardiac Optical Mapping System in Translational Animal Models

**DOI:** 10.3389/fphys.2021.696270

**Published:** 2021-08-20

**Authors:** Manuel Marina-Breysse, Alba García-Escolano, Joaquín Vila-García, Gabriel Reale-Nosei, José M. Alfonso-Almazán, Ping Yan, Jorge G. Quintanilla, Leslie M. Loew, Peter Lee, David Filgueiras-Rama

**Affiliations:** ^1^Centro Nacional de Investigaciones Cardiovasculares (CNIC), Myocardial Pathophysiology Area, Madrid, Spain; ^2^Centro de Investigación Biomédica en Red de Enfermedades Cardiovasculares (CIBERCV), Madrid, Spain; ^3^Escuela Técnica Superior de Ingenieros de Telecomunicación, Universidad Politécnica de Madrid, Madrid, Spain; ^4^Richard D. Berlin Center for Cell Analysis and Modeling, University of Connecticut Health Center, Farmington, CT, United States; ^5^Department of Cardiology, Hospital Clínico San Carlos, Madrid, Spain; ^6^Essel Research and Development Inc., Toronto, ON, Canada

**Keywords:** optical mapping, cardiac electrophysiology, voltage sensitive dyes, calcium sensitive dyes, animal models

## Abstract

Clinicians, biologists, physicists, engineers, and computer scientists are coming together to better understand heart disease, which is currently the leading cause of death globally. Optical mapping, a high-speed fluorescence imaging technique that visualizes and measures key cardiac parameters such as action potentials, cytosolic calcium transients, and fibrillation dynamics, is a core research tool that has arisen from such interdisciplinary collaborations. In an effort to broaden its use, especially among clinical scientists and students, we developed a complete and low-cost optical mapping system, including a constant-flow Langendorff perfusion system, which minimizes the economic threshold to widespread use of this powerful tool in cardiac electrophysiology research. The system described here provides high spatiotemporal resolution data about action potentials, intracellular calcium transients and fibrillation wave dynamics in isolated Langendorff-perfused hearts (pigs and rabbits), relevant for translational research. All system components and software elements are fully disclosed with the aim of increasing the use of this affordable and highly versatile tool among clinicians, basic scientists and students wishing to tackle their own research questions with their own customizable systems.

## Introduction

In cardiac electrophysiology and drug testing, optical mapping is a core tool used at an intermediate experimental stage between basic molecular analyses and the clinic, motivating further investigations at the molecular level and *in vivo*. This becomes more clinically relevant with the use of animal models with translational value (Lee et al., 2017; Quintanilla et al., 2019). At present, optical mapping is a powerful translational research method for probing cellular electrophysiology in whole-heart preparations. This method is based on recording fluorescence changes at high spatiotemporal resolution using voltage and/or calcium sensitive probes (Herron et al., 2012; Lee et al., 2017), for example, that enable the investigation of a broad range of physiological questions (Lee et al., 2012c, 2019; Quintanilla et al., 2019). Although optical mapping is still an evolving method with drawbacks that need to be addressed, it has produced many clinically relevant experimental results (O’shea et al., 2020).

Currently, a complete and fully operational optical mapping system, including all the optical filters and components for a Langendorff perfusion system, is prohibitively expensive for many basic science and clinical research groups. Moreover, clinicians and students new to the field often have limited access to an optical mapping system, typically a shared resource, because damaging components may jeopardize ongoing and future experiments.

In this study, we aimed at developing and validating a low-cost and fully operational optical mapping system that can be implemented and utilized by clinicians, early career scientists and students with limited experience in the field. The cost is at least an order of magnitude lower than that of a conventional optical mapping system combined with a constant-flow Langendorff perfusion system. Fibrillation and drug testing experiments were performed on rabbit and pig hearts to demonstrate its utility for translational cardiac research.

## Materials and Methods

### Optical Mapping System and Components

**FIGURE 1:**
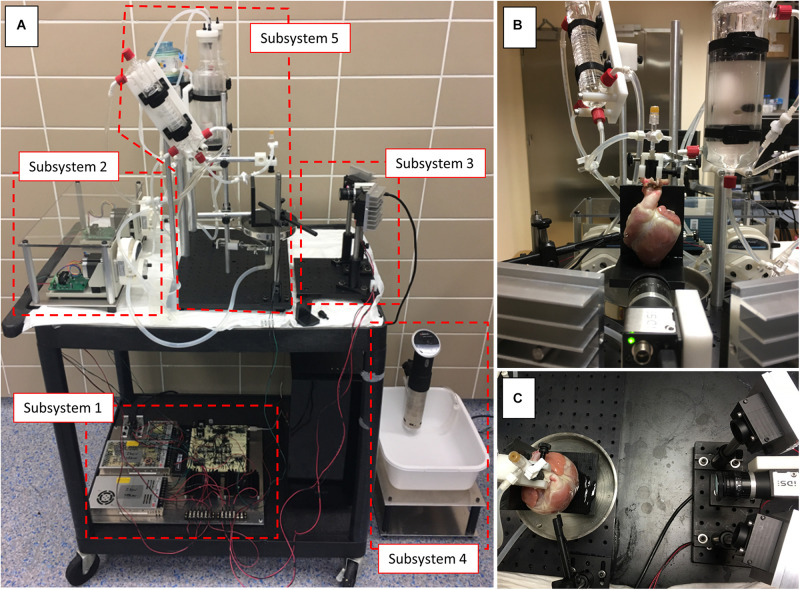
Optical mapping system. **(A)** Complete optical mapping system mounted on a cart. **(B,C)** Different points of view of the system during an optical mapping experiment using a pig heart.

Figure 1 shows the complete optical mapping system, divided into five subsystems as follows:

*Subsystem 1****:*** A custom circuit (Figure 1A, Subsystem 1) was designed to drive two high-power excitation light sources [two red or two green light-emitting diodes (LEDs)], control the two peristaltic pumps on the constant-flow Langendorff perfusion system, and generate biphasic electrical stimuli at variable frequencies, pulse amplitudes and pulse durations. Communication via USB between the circuit board and desktop computer (in our case, running Windows 10 Pro) was made possible with a USB interface module incorporated into the circuit. The circuit can be constructed on a solderless breadboard by a biomedical engineering student, for example, or anyone with experience prototyping circuits on breadboards. Researchers and students with more experience can design a printed circuit board and build an enclosure for all the electronics. Supplementary Figures 1–7 show detailed schematics of the circuit modules, along with a brief description. It should be noted that the peristaltic pumps, LEDs and electric stimulator do not have to be controlled by a computer. A user-interface, though, would have to be designed and implemented to change settings manually (e.g., increase/decrease the excitation light power).

*Subsystem 2****:*** For the constant-flow Langendorff perfusion system, we used two bare-bones peristaltic pumps (Figure 1A, Subsystem 2), one for perfusing the heart at a constant flow-rate and the other for collecting the coronary effluent. The effluent was either directed to a waste bucket or back to a water-jacketed reservoir for recirculation. The flow-rates of these pumps are controllable by a voltage signal ranging from 0 to 10 V. Supplementary Figure 8 shows how to power the pumps and connect them to the circuit board (Subsystem 1).

*Subsystem 3:* The excitation light sources are placed on either side of the high-speed complementary metal-oxide-semiconductor (CMOS) camera for even illumination over the curved heart surface (Figures 1A–C, Subsystem 3). Supplementary Figure 7 shows the wiring diagram for the LEDs. For action potentials and activation wave imaging we used the voltage sensitive dye (VSD) di-4-ANBDQBS and red LEDs (excitation filter RPB630-650) as shown in the schematic of Supplementary Figure 9. Although “first-generation” VSDs such as di-4-ANEPPS and RH-237 can be used (Lee et al., 2017), “second-generation” VSDs such as di-4-ANBDQBS have been shown to produce better signal-to-noise ratios and remain on the plasma membrane for longer durations (Matiukas et al., 2007). For cytosolic calcium transients and calcium wave imaging we used rhod-2AM and green LEDs (excitation filter RPB520-540) as shown in the schematic of Supplementary Figure 9. Emission filters were placed in front of a fast camera lens. Emission filters RPE690LP and RPB560-610 were used for voltage and calcium optical mapping, respectively (Supplementary Figure 9).

**FIGURE 2:**
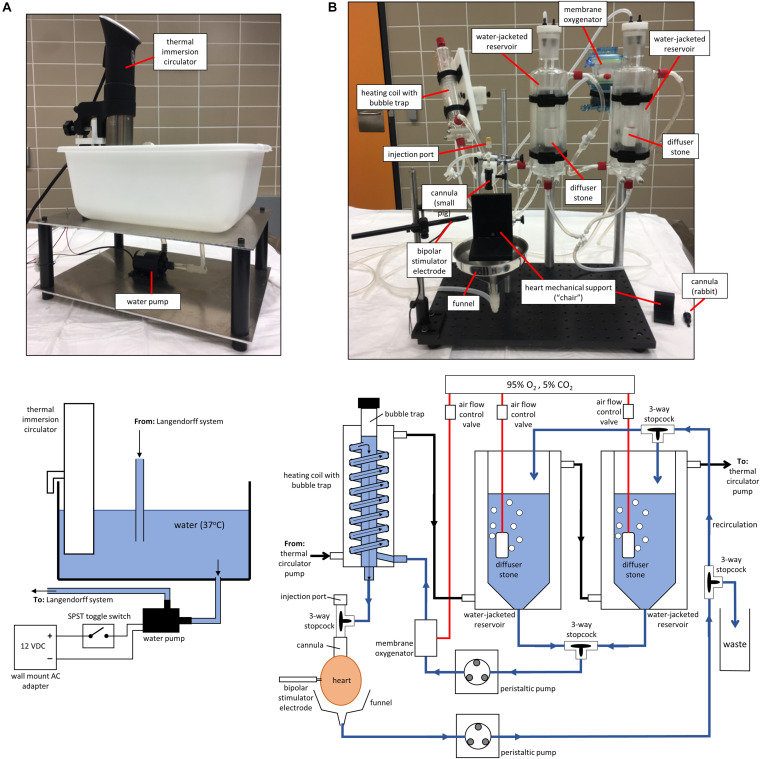
Pictures and schematics of Subsystems 4 and 5. **(A)** Subsystem 4: picture and schematic of the thermal circulator pump. **(B)** Subsystem 5: picture and schematic of the constant-flow Langendorff perfusion system.

*Subsystem 4:* A thermal immersion circulator, used in Sous Vide cooking, was used to keep a water bath at 37°C (Figures 1A, Subsystem 4, 2A). A small hole was drilled at the bottom of the water bath plastic container to connect a tubing line from the water bath to a water pump. The water pump, turned on/off by a toggle switch, provides 37°C water to the heating coil just upstream the heart cannula and the water-jacketed reservoirs holding the perfusate (Figure 2A). Although this ensured that the heart was perfused with 37°C solution, the environment surrounding the heart was at room temperature.

*Subsystem 5:* We built a constant-flow Langendorff perfusion system to accommodate rabbit and pig hearts (Figures 1A, Subsystem 5, 2B). A schematic and picture of this subsystem are shown in Figure 2B. Two water-jacketed reservoirs hold perfusate and a peristaltic pump is used to draw solution from the reservoirs to the heart at a constant flow-rate. If a rabbit heart is used, carbogen (95% O_2_, 5% CO_2_) is delivered to the perfusate via diffuser stones placed inside the reservoirs. For a pig heart, carbogen is delivered to the perfusate via diffusion through thin gas-permeable membranes (i.e., membrane oxygenator) to avoid albumin protein denaturation and solution foaming (note: using an antifoam solution is a low cost alternative to using a membrane oxygenator). Before reaching the heart, the perfusate is passed through a heating coil with a built-in bubble trap to maintain a 37°C temperature and to remove bubbles. Although not used in the validation experiments reported here, using an inline filter (e.g., 20 μm) is recommended. Cannulas were machined in-house using acetal plastic. Voltage dye was delivered through an injection port connected to the cannula and calcium dye was dissolved in the perfusate inside the water-jacketed reservoir.

Coronary effluent is collected using a funnel placed below the heart. Another peristaltic pump transfers this effluent to either a waste bucket or back to the water-jacketed reservoir for recirculation. This pump was set at a flow-rate higher than that of the pump perfusing the heart to avoid overflow in the funnel. With the glassware used in this study, the volume of the recirculating perfusate can range from ∼150 to ∼500 mL, depending on how much solution is placed in the water-jacketed reservoirs. Larger-volume reservoirs can be used to increase the recirculating perfusate volume. A stainless-steel bipolar stimulator electrode was used for focal electrical stimulation of the heart. As shown in Figure 2B, a mechanical support made out of acetal plastic was used to cradle the heart, which prevented hearts from swinging side-to-side during imaging. Although blebbistatin was used as an excitation-contraction uncoupler, it is difficult to completely stop contraction in the atria and ventricles of pig hearts. Finally, we made custom glassware holders in-house with acetal plastic to hold the heating coil and water-jacketed reservoirs. Low-cost lab clamps can be used instead.

Manufacturer part numbers, costs and suppliers are detailed in the Supplementary Material. Although the USB3.0 PCIe card was included in the component price list, we did not include the cost of a desktop computer. Additional detailed descriptions of the low-cost optical mapping system software are also provided in the Supplementary Material.

### Image Acquisition Software

The high-speed CMOS camera used in this study is but one example of relatively low-cost cameras suitable for optical mapping. For the camera used in this study, researchers should download and install the latest IDS Software Suite (IDS Imaging Development Systems GmbH, Obersulm, Germany). The uEye Cockpit, which is one of the applications installed, can be used to configure the camera, align and focus images. Complete details about the camera settings used in this study, custom written C# acquisition software and the MATLAB (The MathWorks Inc., Natick, MA, United States) commands that can be used for image acquisition with ready-to-use DLLs are provided in the Supplementary Material.

### Hardware Control Software

The microcontroller program was written in C within the freely available SimpleIDE (Parallax Inc., Rocklin, CA, United States). The microcontroller used in this study has eight cores, making it much less challenging to execute multiple tasks simultaneously. Although we only used two cores in this study, one can use another core as a temperature controller, for example. The C source code and the MATLAB commands for communicating with the circuit board are provided in the Supplementary Material.

### Langendorff-Perfused Rabbit and Pig Hearts

The studies were conducted in accordance with institutional guidelines and regulations [National (ECC/566/2015, RD53/2013) and European (2010/63/EU) regulation guidelines for the care and use of laboratory animals]. All *in vivo* experimental procedures were evaluated and granted by the Institutional Animal Care and Use Committee (IACUC) of CNIC and the Local Competent Authority.

Three female New Zealand White rabbits (∼3 kg) and two Large White male pigs (∼30 kg) were used in this study. Surgical procedures were performed under general anesthesia following premedication with a combination of intramuscular Xylazine (10 mg/kg i.m.) and Midazolam (2 mg/kg i.m.) and induced with a slow intravenous administration of Ketamine (20 mg/kg i.v.). After intubation and venous catheterization through the marginal vein, animals were mechanically ventilated with intermittent positive pressure and anesthesia was maintained by a combination of Fentanyl (0.010 mg/kg/h i.v.) and Sevoflurane (2%). To avoid coronary blood coagulation, Heparin (300 UI/kg) was administered and vital signs were monitored during the procedure. Median sternotomy was performed to expose the heart and a 9 VDC battery was used to induce ventricular fibrillation during the extraction to minimize the chance of air bubbles entering the coronary arteries. Excised hearts were then submerged in cold (4°C) Tyrode’s solution, cleaned and then cannulised through the aorta. The isolated hearts were then connected to the constant-flow Langendorff-perfusion system described earlier in this text. Hearts were perfused with oxygenated (95% O_2_, 5% CO_2_) modified Tyrode’s solution (composition in mM: NaCl 130, NaHCO_3_ 24.2, NaH_2_PO_4_ 1.2, MgCl_2_ 0.6, KCl 4.7, Glucose 12, and CaCl_2_ 2.2) at a circulating flow-rate of 30 mL/min for rabbit hearts and Tyrode’s solution (composition in mM: NaCl 130, NaHCO_3_ 24, NaH_2_PO_4_ 1.2, MgCl_2_ 1, KCl 4, Glucose 5.6, CaCl_2_ 1.8, and albumin 0.04 g/L) at a circulating flow-rate of 200–240 mL/min for pig hearts. Ionic pH of the perfusate (7.4) and temperature (36–37°C) were monitored during the experiment. Ventricular fibrillation was induced by touching the epicardium with a 9 VDC battery or by rapidly pacing the ventricles.

### Dye Loading and Drug Action Effect Study

For voltage imaging in pig hearts, hearts were loaded with 500 μL of di-4-ANBDQBS stock solution (10 mg of di-4-ANBDQBS dissolved in 3 mL of pure ethanol) diluted in 10 mL of Tyrode’s solution. This dye solution was delivered slowly over a 1-min period through an injection port connected to the cannula. After voltage dye loading, the heart was perfused with Tyrode’s solution containing 10 μM blebbistatin (part #: 674289-55-5; Cayman Chemical Company, Ann Arbor, MI, United States) to minimize contraction.

For voltage imaging in rabbit hearts, hearts were loaded with 25 μL of di-4-ANBDQBS stock solution (10 mg of di-4-ANBDQBS dissolved in 3 mL of pure ethanol) through an injection port connected to the cannula over a 1-min period. For calcium imaging in rabbit hearts, rhod-2AM stock solution (1 mg/mL in DMSO; part #: 50024, Biotium Inc., Hayward, CA, United States) was added to the perfusate in the water-jacketed reservoir to achieve a dye concentration of 10 μM. The perfusate volume in the system was reduced to ∼150 mL before adding rhod-2AM to minimize dye usage and the heart was perfused with recirculation for 45 min. After voltage/calcium dye loading, the heart was perfused with solution containing 10 μM blebbistatin to minimize contraction. For the drug effect experiments, solution containing the drug under investigation (2 μM nifedipine, N7634; Sigma-Aldrich Dorset, United Kingdom/1 μM flecainide, F6777; Sigma-Aldrich Dorset/10 nM isoproterenol, Sandoz International GmbH, Germany) was placed in one of the water-jacketed reservoirs and the heart was perfused, without recirculation, with this solution for ∼5 min before imaging.

### Data Processing and Analysis

**FIGURE 3:**
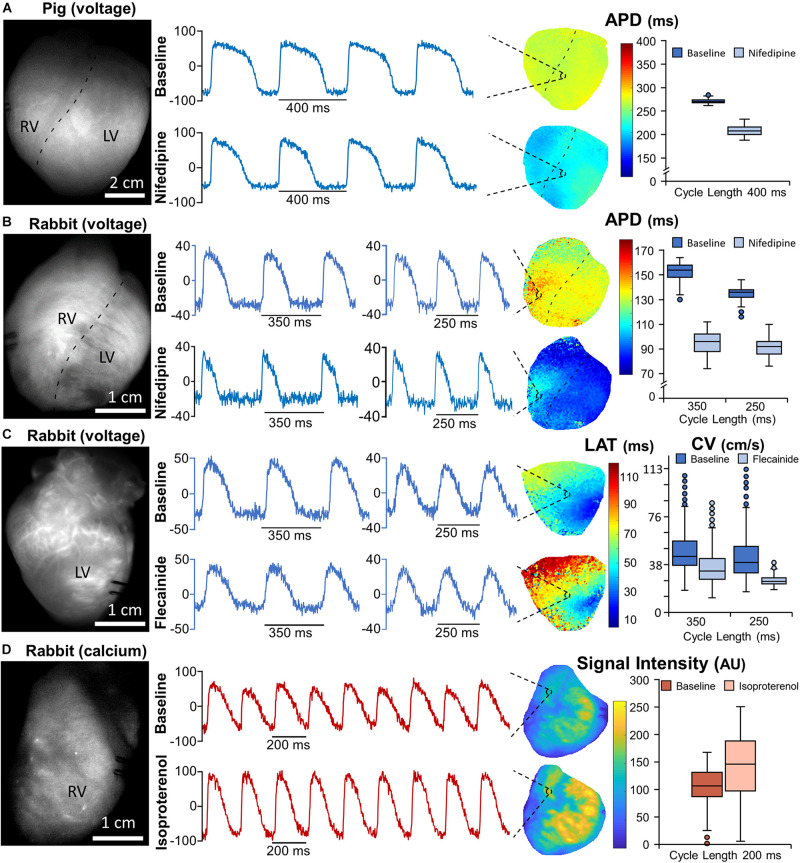
Optical mapping recordings using voltage and calcium sensitive dyes. **(A,B)** Baseline fluorescence images of sample pig **(A)** and rabbit **(B)** hearts after loading with voltage sensitive dye (VSD) di-4-ANBDQBS **(left panels)**. Normalized fluorescence signals, at 400 ms pacing cycle length (pig heart) and 350 and 250 ms pacing cycle lengths (rabbit heart), from representative pixels on the action potential duration (APD) maps at baseline **(upper map)** and after nifedipine administration **(lower map; middle panels)**. Quantification of APD90 at baseline and after nifedipine administration in the pig and rabbit hearts **(right panels)**. **(C)** Baseline fluorescence image of a second rabbit heart after loading with VSD di-4-ANBDQBS **(left panel)**. Normalized fluorescence signals at 350 and 250 ms pacing cycle lengths from a representative pixel on the local activation time (LAT) map at baseline **(upper map)** and after flecainide administration **(lower map; middle panels)**. Quantification of conduction velocity (CV) at baseline and after flecainide administration **(right panel)**. **(D)** Baseline fluorescence image of a third rabbit heart after loading with calcium-sensitive dye rhod-2AM **(left panel)**. Normalized fluorescence signals of calcium transients at 200 ms pacing cycle length from a representative pixel on the signal intensity map at baseline **(upper map)** and after isoproterenol administration **(lower map; middle panels)**. All signals are from spatially averaged pixels (3 × 3 pixels). All maps shown used spatial and temporal filtering (3 × 3 to 5 × 5 pixels and third-order median filtering, respectively). The boxplots show data from each respective map considering values between the 1st and 99th percentile. LV, left ventricle; RV, right ventricle.

**FIGURE 4:**
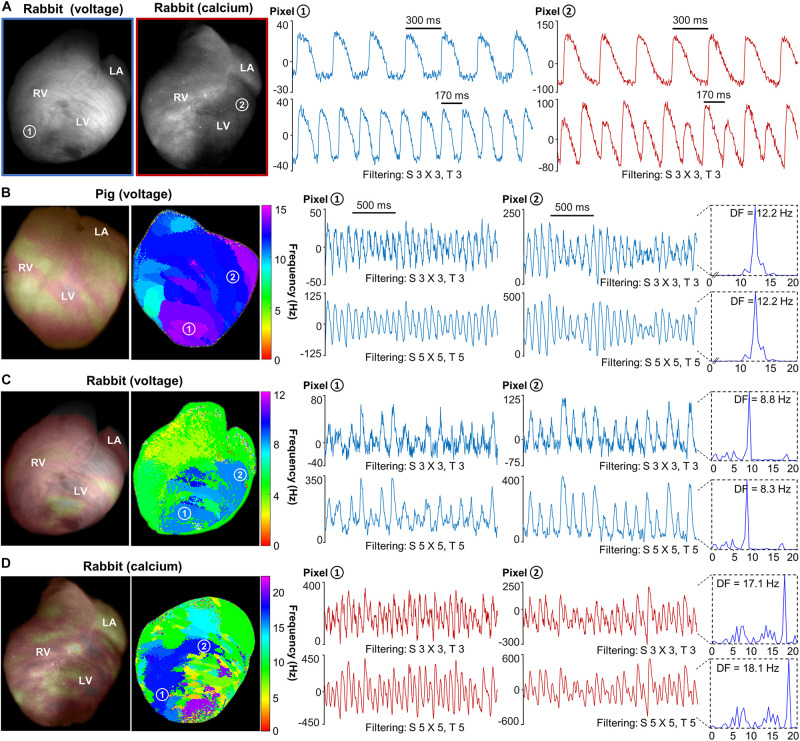
Optical mapping of voltage and calcium alternans and ventricular fibrillation. **(A)** Baseline fluorescence image of sample rabbit hearts after dye loading with di-4-ANBDQBS (voltage) and rhod-2AM (calcium), respectively **(two leftmost panels)**. Normalized fluorescence signals showing action potential and calcium transient alternans at 300 and 170 ms pacing cycle lengths from representative pixels on the rabbit hearts. **(B)** Normalized fluorescence snapshot of a pig heart at one time point during ventricular fibrillation (post-optimization, bright/dark areas represent depolarized/repolarized tissue, respectively; arbitrary units) and associated dominant frequency (DF) map of a 5-s-long recording **(two leftmost panels)**. Normalized fluorescence signals of action potentials during ventricular fibrillation and associated DF values from pixels 1 and 2. **(C)** Normalized fluorescence snapshot of a rabbit heart at one time point during ventricular fibrillation and associated DF map of a 5-s-long recording **(two leftmost panels)**. Normalized fluorescence signals of action potentials during ventricular fibrillation under flecainide effect and associated DF values from pixels 1 to 2. **(D)** Normalized fluorescence snapshot of a rabbit heart at one time point during ventricular fibrillation and associated DF map of a 5-s-long recording **(two leftmost panels)**. Normalized fluorescence signals of calcium transients during ventricular fibrillation and associated DF values from pixels 1 to 2. Blue and red color signals show optical action potentials and calcium transients, respectively. Two different spatial (S) and temporal (T) filtering are shown as indicated in panels **(B–D)**. LA, left atrium; LV, left ventricle; RV, right ventricle.

Custom analysis software was written in MATLAB to generate the data presented in Figures 3,4 and the Supplementary Movies. Conduction velocity (CV) was estimated according to a polynomial fitting method as described elsewhere (Bayly et al., 1998). Briefly, spatial coordinates (*X*, *Y*) and activation times (*t*) were adjusted to a cubic spline surface, whose gradient provided the local direction and magnitude of CV. Action potential duration (APD) maps were calculated as the difference between times at maximum ascending slope (activation) and 90% of repolarization for each pixel. A set of basic image processing MATLAB programs, along with sample rabbit and pig heart data sets, are provided as a starting point (Supplementary Material). Open-source optical mapping analysis software packages can be found elsewhere (Laughner et al., 2012; Li et al., 2019; O’shea et al., 2019; Tomek et al., 2021).

## Results

### Optical Mapping System

A fully operational optical mapping system, including a constant-flow Langendorff perfusion system, was designed and built at a cost just under $5,000 USD (Figure 1). A team of three non-experts on optical mapping, comprising a clinical cardiologist, a medical student and a biomedical engineering student, were able to assemble and use the complete system. While cardiac surgery for heart excision and aortic cannulation for perfusion was performed only by the clinical cardiologist, all team members were able to run experiments and analyze the data.

### Optical Mapping of Voltage and Calcium Dynamics During Organized Rhythms

Optical mapping of transmembrane voltage was performed in two rabbit and two pig hearts, while optical mapping of free intracellular calcium transients was performed in one rabbit heart. Small and often hardly noticeable changes with the naked eye in the baseline fluorescence of the movies after dye loading reflect the time course of transmembrane potential (voltage) and intracellular calcium (calcium transients). Therefore, removal of the fluorescence baseline and inversion (the latter only for voltage signals) is required to optimize their visualization. Baseline (pre-optimization) fluorescence snapshots were displayed in grayscale (Figure 3). Normalized fluorescence snapshots/movies after optimization were displayed in reddish tones superimposed on the cardiac anatomy (Figure 4). In the latter, bright/dark regions correspond to depolarized/repolarized tissue, respectively.

Cardiac electrophysiology was altered using three different drugs, specifically changing the APD, CV and intracellular calcium transient amplitudes. Optical mapping of transmembrane voltage changes during cardiac pacing and nifedipine administration showed a marked decrease in APD90 in both rabbit and pig hearts (Figures 3A,B). Flecainide, on the other hand, significantly decreased the CV throughout the mapping surface (Figure 3C). Although we did not aim to provide a complete assessment of APD and CV values in a large series of animals, our results on CV and APD are in agreement with previous reports from our group and others using the same animal species (Pandit et al., 2011; Lee et al., 2017, 2019; Kappadan et al., 2020). Optical mapping of intracellular calcium transients at baseline and after isoproterenol administration showed an overt increase in calcium transient amplitudes and the disappearance of alternans (Figure 3D).

Cardiac alternans is a phenomenon in which there is a beat-to-beat oscillation in contraction strength, and action potential and calcium transient profiles, and its significance in heterogeneous cardiac impulse propagation and cardiac fibrillation risk has been studied extensively (Uzelac et al., 2017). Figure 4A demonstrates this phenomenon in the rabbit heart during pacing at 170 ms CL. In contrast, alternants were not present during pacing at 300 ms CL.

### Optical Mapping of Voltage and Calcium Dynamics During Ventricular Fibrillation

Optical mapping of cardiac fibrillation dynamics using transmembrane voltage imaging was one of the most challenging tests for our low-cost system because of the significantly smaller optical signals and fast activation rates. As demonstrated in Figures 4B,C, the system was able to generate accurate dominant frequency (DF) maps in both the pig and rabbit hearts. DF maps were also generated from intracellular calcium imaging during VF in the rabbit heart (Figure 4D). The Supplementary Movies show activation wave dynamics during paced and fibrillatory activity in pig and rabbit hearts.

## Discussion

We have described a complete and fully operational low-cost optical mapping system that can be implemented and used by clinicians, students, and scientists without expertise in the field. The system is customizable, portable, and capable of providing high spatiotemporal resolution information about action potentials, intracellular calcium transients and fibrillation wave dynamics in two relevant animals models for translational research (Wang et al., 2014; Quintanilla et al., 2019). With an overall cost of just under $5,000 USD, we believe the economic threshold to widespread use of this powerful tool in cardiac electrophysiology research has been eliminated. For reference, buying off-the-shelf components just to build a constant-flow Langendorff perfusion system typically costs over $5,000 USD. In addition, this system can be assembled and used in an undergraduate biomedical engineering laboratory course. Such a course component could expose students to wet-lab work, experimental-based mathematical modeling, image processing, electronics and fluorescence imaging, all important aspects of biomedical engineering. Open-source optical mapping analysis software packages have also been developed by several research groups, which can be used as references for those writing their own software or as primary analysis tools for end-users (Laughner et al., 2012; Li et al., 2019; O’shea et al., 2019; Tomek et al., 2021). However, it should be noted that the costs of the computer, MATLAB software, and key optical mapping chemicals (dyes and blebbistatin) were not included in the system cost. The costs of chemicals and costs associated with animal work will certainly impact the number of experiments that can be performed on a limited budget.

Drastically lowering the cost of experimental tools generally increases experimental data output because of more widespread use. Fields like computational modeling are often limited by a lack of experimental data that provides parameters for computational models or validates any hypotheses generated. Moreover, complex cardiac arrhythmias are a spatiotemporal phenomenon governed by multiple interconnected factors (e.g., calcium and voltage spatial alternans or heterogeneous CV) which are difficult to simulate without relevant experimental data (Boyle et al., 2019). Our hope is that computational research groups can more easily run customized and dedicated optical mapping experiments for generating more realistic models and testing hypotheses. This would lead to new opportunities for integrating experimental data with theory, which is often lacking in the cardiac electrophysiology community and more generally, in the biology community (Goldstein, 2018). Furthermore, the costs and skills required for using smaller translational models, like rabbit hearts, are accessible to many research groups.

Although this system was designed to be used with translational animal models of interest to clinical scientists, optical mapping is also used in smaller animal models (Lee et al.,2012b,d), like mouse/rat hearts, and tissue constructs or monolayers using human induced pluripotent stem cell-derived cardiomyocytes (Herron et al., 2012; Lee et al., 2012a; Climent et al., 2015). Higher camera sensitivity, a temperature-controlled environment and precision fluid-handling are required for such preparations. Improvements to the system described here may include implementing a temperature-controlled environment and ECG measurement electronics synchronized with optical mapping recordings. But performing optical mapping in mouse/rat hearts and tissue constructs or monolayers requires a high-speed camera with higher sensitivity and lower noise. Most current high-speed CMOS cameras have lower light sensitivity and higher noise than cameras typically used by the optical mapping community, which is the biggest limitation of the system described in this study (Lee et al., 2017). Fortunately, CMOS image sensors are constantly improving in response to a growing demand for higher speed and higher sensitivity cameras for industrial and scientific applications. Therefore, the design of a tissue construct or monolayer low-cost optical mapping system may be feasible with new generation CMOS image sensor technology in the near future.

## Data Availability Statement

The original contributions presented in the study are included in the article/Supplementary Material, further inquiries can be directed to the corresponding authors.

## Ethics Statement

The animal study was reviewed and approved by the Institutional Animal Care and Use Committee (IACUC) of CNIC and the Local Competent Authority.

## Author Contributions

MM-B, AG-E, PL, and DF-R conceived and designed the research. MM-B, AG-E, JV-G, GR-N, JA-A, JQ, PL, and DF-R performed the experiments and analyzed the data. MM-B, AG-E, JV-G, GR-N, JA-A, PY, JQ, LL, PL, and DF-R drafted, edited, and revised the manuscript. All authors contributed to the article and approved the submitted version.

## Conflict of Interest

PL is both an owner and employee of Essel Research and Development Inc. PY is both an owner and employee. LL is an owner of Potentiometric Probes, LLC. The remaining authors declare that the research was conducted in the absence of any commercial or financial relationships that could be construed as a potential conflict of interest.

## Publisher’s Note

All claims expressed in this article are solely those of the authors and do not necessarily represent those of their affiliated organizations, or those of the publisher, the editors and the reviewers. Any product that may be evaluated in this article, or claim that may be made by its manufacturer, is not guaranteed or endorsed by the publisher.
