# Supplementary material for: A Complete and Low-Cost Cardiac Optical Mapping System in Translational Animal Models
Source: Front Physiol. 2021 Aug 20;12:696270. doi: 10.3389/fphys.2021.696270 (PMC8417781; doi:10.3389/fphys.2021.696270)
Supplement: Supplementary file 5 [file Data_Sheet_2.docx]

**SUPPLEMENTARY MATERIAL**


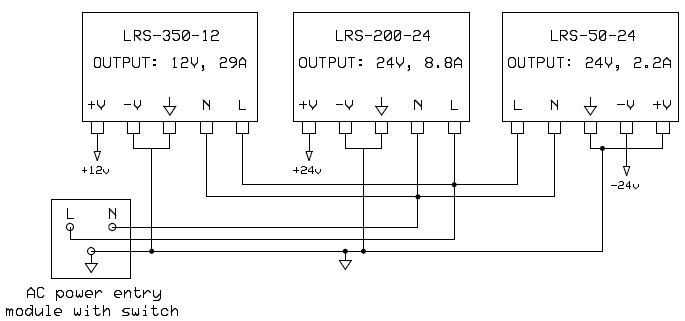


**Suppl. Figure 1. Circuit schematic 1:** Power supplies for the circuit board, LEDs, peristaltic pumps and electrical stimulator.


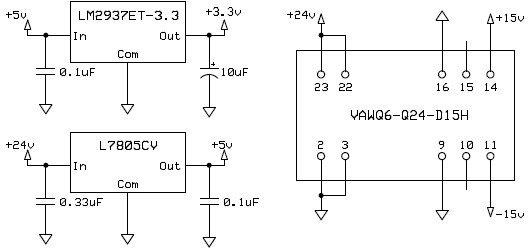


**Suppl. Figure 2.** **Circuit schematic 2:** Three voltage regulators outputting +3.3V, +5V and ±15V. These voltage supplies are needed to power the other components on the circuit board.


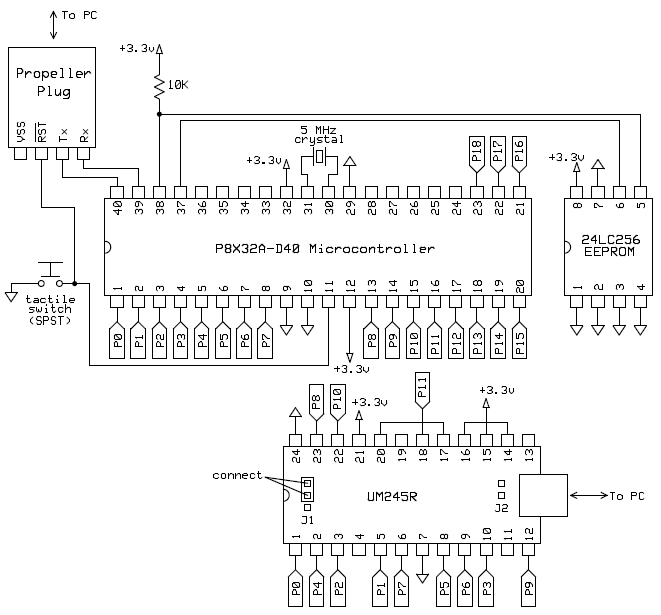


**Suppl. Figure 3.** **Circuit schematic 3:** Microcontroller and USB interface module. The microcontroller has 8 cores, enabling multiple tasks to be run simultaneously in a straightforward manner. The *Propeller Plug* is used to program the microcontroller. The program is stored in the EEPROM, so the *Propeller Plug* can be disconnected from the computer and removed from the circuit board after uploading the microcontroller program.


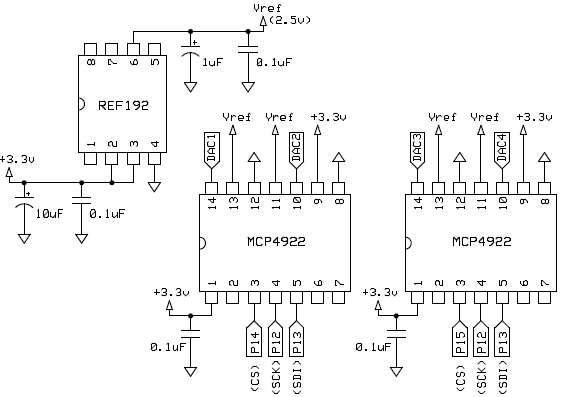


**Suppl. Figure 4.** **Circuit schematic 4:** Four digital-to-analog converters for controlling the excitation light power, two peristaltic pump flow-rates and electrical stimulation pulse amplitude.


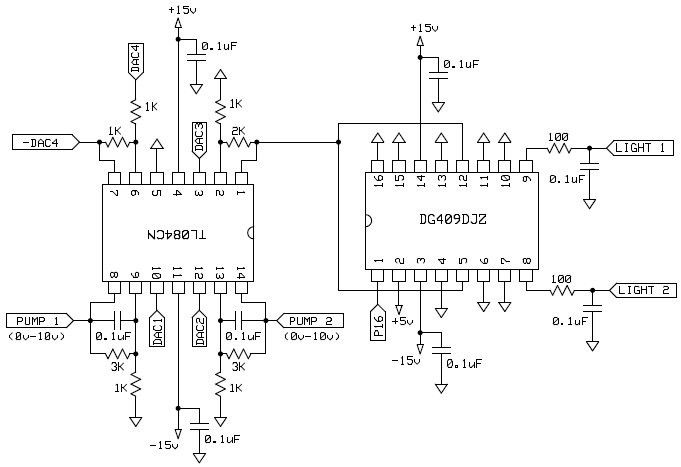


**Suppl. Figure 5.** **Circuit schematic 5:** Analog signal conditioning and control circuitry.


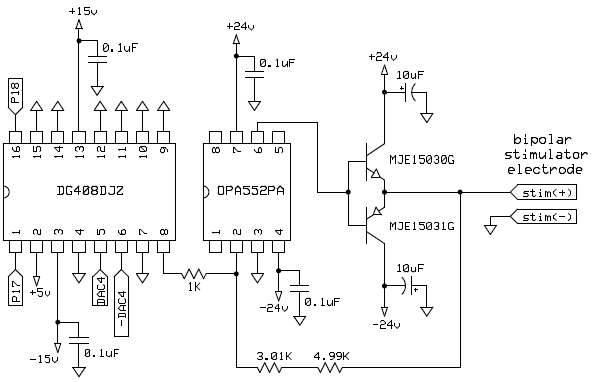


**Suppl. Figure 6. Circuit schematic 6:** Analog signal conditioning and control circuitry.


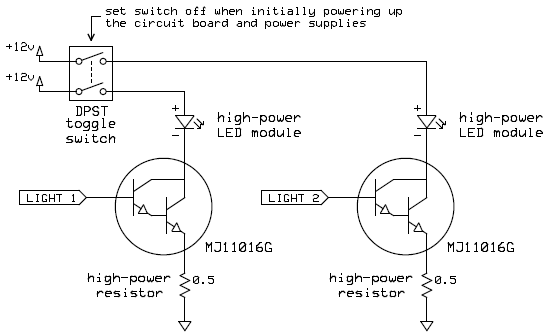


**Suppl. Figure 7. Circuit schematic 7:** LED drive and wiring diagram. The LEDs and Darlington transistors (MJ11016G) must be mounted on heatsinks to dissipate heat. Depending on the LED drive currents used, larger heatsinks and/or fans may be needed to enhance heat dissipation.

**
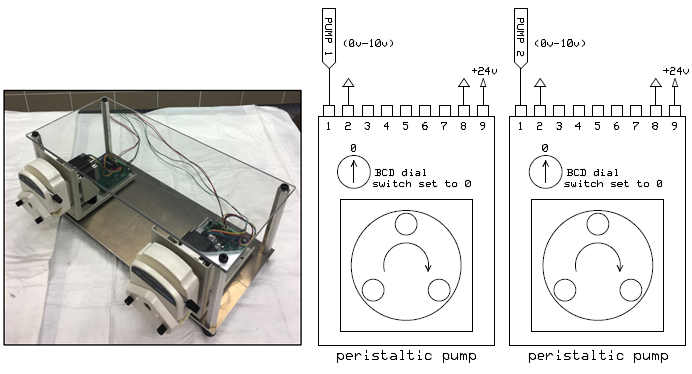
**

**Suppl. Figure 8. Subsystem 2: Peristaltic pumps.** Wiring diagram and settings for the two bare-bones peristaltic pumps.

**
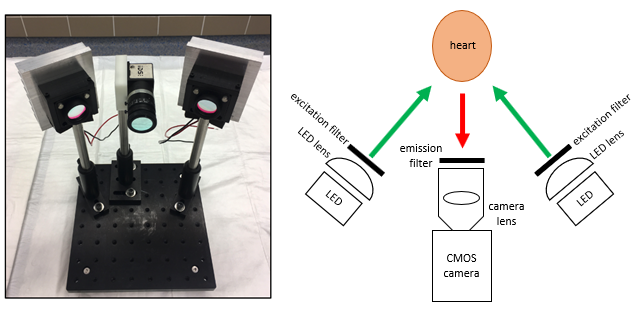
**

**Suppl. Figure 9. Subsystem 3: Fluorescence imaging camera and excitation light sources.** Fluorescence excitation and imaging subsystem.

**Optical mapping system: components and price lists**

**Manufacturer Part # (Source) Description Price (USD) Quantity**

WBU-208-R (1) 3220-point solderless breadboard 35.95 1

**Subtotal: 35.95**

**Suppl. Figure 1**

LRS-350-12 (2) Switching power supply (12V, 29A) 34.21 1

LRS-200-24 (2) Switching power supply (24V, 8.8A) 25.71 1

LRS-50-24 (2) Switching power supply (24V, 2.2A) 11.80 1

DC11.0001.001 (2) AC power entry module with switch 10.34 1

**Subtotal: 82.06**

**Suppl. Figure 2**

LM2937ET-3.3/NOPB (2) 3.3V voltage regulator 1.69 1

SR201C104KAR (2) 0.1uF ceramic capacitor 0.32 2

ECA-2AM100 (2) 10uF electrolytic capacitor 0.23 1

L7805CV (2) 5V voltage regulator 0.50 1

RDER71H334K1M1H03A (2) 0.33uF ceramic capacitor 0.53 1

VAWQ6-Q24-D15H (2) DC/DC converter +15/-15 Vdc 27.41 1

**Subtotal: 31.00**

**Suppl. Figure 3**

KSA0M211LFTR (2) Tactile switch (SPST) 0.57 1

MFR-25FBF52-10K (2) 10 kOhm resistor 0.13 1

ECS-50-20-4X (2) 5MHz crystal 0.69 1

P8X32A-D40 (2) 32-bit multi-core microcontroller 7.99 1

24LC256-I/P (2) 256 kbit EEPROM 0.82 1

32201 (2) Microcontroller programming module 14.99 1

(Propeller Plug)

UM245R (2) USB interface module 20.00 1

AK672/2-3 (3) USB A-B male cable (3.00m) 3.30 1

**Subtotal: 48.49**

**Suppl. Figure 4**

SR201C104KAR (2) 0.1uF ceramic capacitor 0.32 4

ECA-2AM100 (2) 10uF electrolytic capacitor 0.23 1

T350A105K035AT (3) 1uF tantalum capacitor 0.76 1

REF192GPZ (2) Voltage reference (2.5V) 5.06 1

MCP4922-E/P (2) DAC Dual 12-bit 2.62 2

**Subtotal: 12.57**

**Suppl. Figure 5**

TL084CN (2) Operational amplifier (4 channel) 0.55 1

DG409DJZ (2) 4:1 analog multiplexer (2 channel) 2.68 1

SR201C104KAR (2) 0.1uF ceramic capacitor 0.32 8

MFR-25FBF52-1K (2) 1 kOhm resistor 0.10 5

MFR-25FTE52-3K (2) 3 kOhm resistor 0.12 2

MFR-25FBF52-2K (2) 2 kOhm resistor 0.10 1

MFR-25FBF52-100R (2) 100 Ohm resistor 0.10 2

**Subtotal: 6.83**

**Suppl. Figure 6**

DG408DJZ (3) 8:1 analog multiplexer (1 channel) 2.75 1

OPA552PA (3) Operational amplifier (1 channel) 4.69 1

MJE15030G (2) Bipolar transistor NPN 1.50 1

MJE15031G (2) Bipolar transistor PNP 1.50 1

SR201C104KAR (2) 0.1uF ceramic capacitor 0.32 4

ECA-2AM100 (2) 10uF electrolytic capacitor 0.23 2

MFR-25FBF52-1K (2) 1 kOhm resistor 0.10 1

MFR-25FBF52-3K01 (2) 3.01 kOhm resistor 0.10 1

MFR-25FBF52-4K99 (2) 4.99 kOhm resistor 0.10 1

**Subtotal: 12.48**

**Suppl. Figure 7**

ST242D00 (2) DPST toggle switch 4.92 1

CBT-90-G-L11-CM101 (2) Green LED module 77.50 2

CBT-90-RX-L15-BN101 (2) Red LED module 86.74 2

L003 (4) 3.945" wide aluminum heatsink (3" length) 11.58 2

I003 (4) 3.500" wide aluminum heatsink (3" length) 4.08 2

MJ11016G (2) NPN Darlington transistor (TO-3 package) 7.23 2

641A (2) TO-3 heatsink for Darlington transistor 10.90 2

HS50 R50 F (2) 0.5 Ohm 50W resistor 4.72 2

** The heatsinks are for the LEDs and Darlington transistors*

**Subtotal: 410.42**

**Suppl. Figure 8**

T100-S18 (5) Barebones peristaltic pump (0-10V control) 228.00 2

3 roller pump head

**Subtotal: 456.00**

**Suppl. Figure 9**

RPB560-610 (6) 585BP50 RapidBand optical filter 175.00 1

RPB520-540 (6) 530BP20 RapidBand optical filter 175.00 2

RPE690LP (6) 690LP RapidEdge optical filter 100.00 1

RPB630-650 (6) 640BP20 RapidBand optical filter 175.00 2

LA1951 (7) Plano-convex lens for LED 25.14 2

CP33T (7) 0.50” thick cage plate (LA1951 lens holder) 23.27 2

SM1L03 (7) 0.30” lens tube (excitation filter holder) 12.52 2

DF6HA-1S (8) 1/2" camera lens (F1.2, 6mm) 119.00 1

SM1A36 (7) M27x0.5 adapter (emission filter holder) 21.86 1

UI-3360CP-NIR-GL (9) High-speed CMOS camera 1,260.00 1

IOI-E4-USB3-4P-4HS (9) 4-Port USB3.0 PCIe card 150.00 1

TR6 (7) 1/2" optical post, 6” length 7.33 3

PH3 (7) 1/2" post holder, 3” length 8.52 3

BA1 (7) 1” x 3” mounting base 5.77 2

BA2 (7) 2” x 3” mounting base 7.52 1

MB8 (7) 8” x 8” aluminum breadboard 77.22 1

**Subtotal: 2791.55**

**Figure 2A**

Precision Cooker Nano (10) Thermal immersion circulator 129.00 1

EWP-D401250 (11) 8.3 L/min water pump 18.50 1

10TC220 (2) SPST toggle switch 2.31 1

TRE25R120-12G03-Level-VI (2) Wall mount AC adapter (12VDC output) 15.13 1

**Subtotal: 164.94**

**Figure 2B**

Custom glassware (12) Heating coil with bubble trap 110.00 1

Custom glassware (12) Water-jacketed reservoir (~ 1/3 L) 130.00 2

** Similar glassware can be purchased off-the-shelf*

MB1218 (7) 12” x 18” aluminum breadboard 193.64 1

TR12 (7) 1/2" optical post, 12” length 11.37 1

SWC (7) Rotating clamp for 1/2" post 25.00 1

PH3 (7) 1/2" post holder, 3” length 8.52 1

BA2 (7) 2” x 3” mounting base 7.52 1

**Bipolar stimulator electrode**

1003 (2) Pig-tail cable, 0.1” 2-pin (4 pack) 1.95 1

92705K12 (13) 316 stainless steel wire (0.02” diameter) 20.45 1

** Tubing, valves, 3-way stopcocks, etc. are not included in this listing as they are found in most experimental physiology labs*

** Although the glassware holders, cannula, heart mechanical support, etc. were built in-house, suitable low-cost off-the-shelf components can be used instead*

** We obtained the membrane oxygenator as sample material from the Hospital Clínico San Carlos and purchased the diffuser stones at a local aquarium supply shop.*

**Subtotal: 638.45**

**Grand total: 4690.74**

**Note: Prices may vary since those shown here are based on the time of purchase.**

**Sources for parts:**

(1) Jameco Electronics

1355 Shoreway Road

Belmont, CA 94002, USA

(2) Mouser Electronics

1000 North Main Street

Mansfield, TX 76063, USA

(3) Digi-Key Electronics

701 Brooks Avenue South

Thief River Falls, MN 56701, USA

(4) HeatsinkUSA, LLC

801 Industrial Park Dr.

Greenville, MI 48838, USA

(5) Longer Precision Pump Co., Ltd.

3rd/4th Floor, Building 6B, University Science Park Baoding National, High - Tech Industrial Development Zone Baoding, Hebei, China

(6) Omega Optical, LLC

21 Omega Drive

Brattleboro, VT 05301, USA

(7) Thorlabs Inc.

56 Sparta Avenue

Newton, NJ 07860, USA

(8) RMA Electronics, Inc.

35 Pond Park Road. Unit #12

Hingham, MA 02043, USA

(9) 1stVision, Inc.

2 Dundee Dr.

Andover, MA 01810, USA

(10) Anova Culinary, Inc.

667 Howard Street

San Francisco, CA 94105, USA

(11) LightObject

21 Blue Sky Court

Sacramento, CA 95828, USA

(12) ProScience (Glass Shop Division)

770 Birchmount Road, Unit #25

Scarborough, ON M1K5H3, Canada

(13) McMaster-Carr Supply Company

600 N County Line Rd.

Elmhurst, IL 60126, USA

**Image acquisition software**

For the camera used in this study, researchers should download and install the latest IDS Software Suite (IDS Imaging Development Systems GmbH, Obersulm, Germany). The uEye Cockpit, which is one of the applications installed, can be used to configure the camera, align and focus images. Below are the settings used in this study:


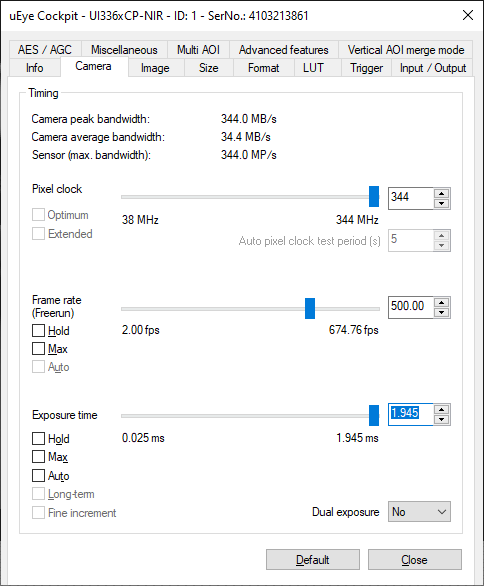


For rabbit and pig hearts, a frame rate of 500 fps is sufficient to capture physiologically relevant action potential and calcium transient dynamics under sinus rhythm and fibrillation.

Reference:

Lee P, Calvo CJ, Alfonso-Almazan JM et al. Low-Cost Optical Mapping Systems for Panoramic Imaging of Complex Arrhythmias and Drug-Action in Translational Heart Models. Scientific reports 2017;7:43217.


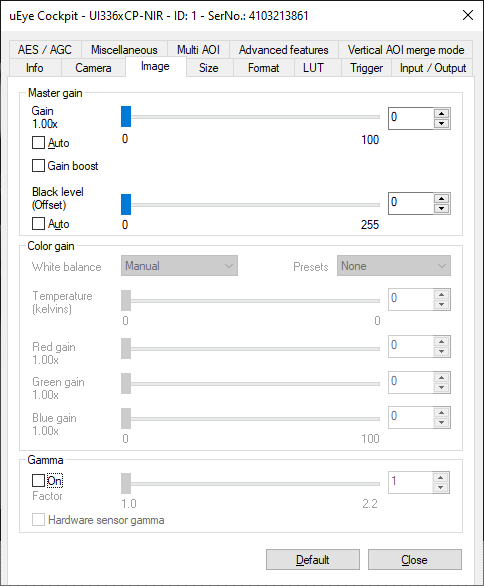


Dye loading results differ from experiment to experiment, resulting in varying fluorescence emission levels. To increase the sensitivity of the camera, the researcher can either set the *Gain* to a value higher than 0 or select the *Gain boost* option. For this study, we used the *Gain boost* option to increase camera sensitivity for some experiments.


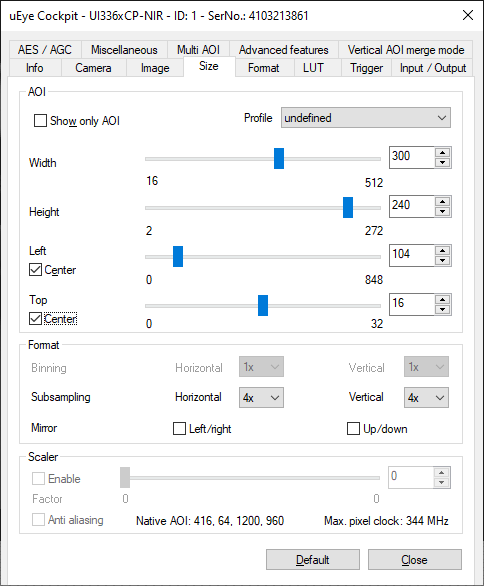


Higher frame rates can be achieved by binning/subsampling and reducing the AOI. The spatial resolution attained by using 300 x 240 pixels was sufficient to capture fibrillation dynamics. To date, most published optical mapping studies use cameras with less than 200 x 200 pixel resolution. This camera did not have a binning option, which usually yields higher signal-to-noise ratios because charges from a group of adjacent pixels are summed/averaged and reported out as a single pixel. Subsampling skips multiple sensor pixels when reading out the image data, so there is no spatial averaging and, therefore, no improvement in the signal-to-noise ratios. Next generation low-cost cameras might provide higher sensitivity and a binning option for further development.

Reference:

Herron TJ, Lee P, Jalife J. Optical imaging of voltage and calcium in cardiac cells & tissues. Circ Res 2012;110:609-23.


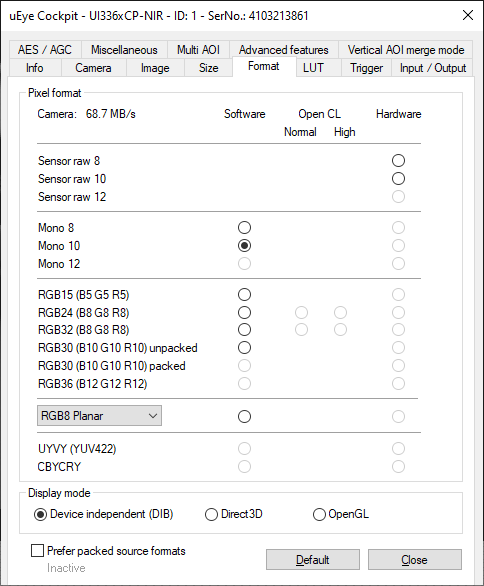


For camera software development, we used the freely-available Microsoft Visual Studio Community 2017 IDE. We wrote a .NET assembly that can be used within MATLAB (The MathWorks Inc., Natick, MA). Researchers and students with experience developing software using the C# programming language can use the provided source code to write all the image acquisition and hardware control software in C#, which may provide better speed performance.

Source code (compressed): Camera_IDS_UI3360CPNIR

Ready-to-use DLLs: Camera_IDS_UI3360CPNIR.dll

uEyeDotNet.dll

**MATLAB commands for image acquisition**

1. Copy the following assemblies to your MATLAB working folder:

Camera_IDS_UI3360CPNIR.dll

uEyeDotNet.dll

1. Make the relevant .NET assembly visible to MATLAB:

asm = NET.addAssembly('C:\Optical Mapping\MATLAB Programs\Camera_IDS_UI3360CPNIR.dll')

1. After creating a folder to hold the image sequence, specify the file name format:

filename = 'D:\OM_Experiment1\frame'; % set file name

(example image file generated: D:\OM_Experiment1\frame13.png)

1. Acquire an image sequence using the *Acquire* static method in the *Camera* class:

% Acquire(number of frames, frame rate (fps), gain boost?, file name)

% Results(1): camera ID

% Results(2): recording time in milliseconds

Results = Camera_IDS_UI3360CPNIR.Camera.Acquire(2500,500,false,filename)

Note: 1) Since all the images are temporarily stored in RAM before saving to a hard drive, be mindful of the number of frames specified

2) The maximum frame rate with the camera setting used is ~670 fps

3) If a frame is dropped during acquisition (rare occurrence), that image file will be missing in the folder. Interpolation can be used to generate the missing frame(s). For example, if frame1031.png is missing, you can generate frame1031.png by taking the average of frame1030.png and frame1032.png.

1. The following provided MATLAB programs can be used as a basic starting point for image processing:

**Note: One of the sample data sets provided was used generate the figures shown below (data set ‘Rabbit Voltage Pacing’).**

**OpticalMapProcess1.m**

% Read image file data into imageStack 3D matrix

clear imageStack

clc;

n = 1500; % number of frames

nrows = 300; % number of vertical pixels

ncols = 240; % number of horizontal pixels

imageStack = double(zeros(nrows,ncols,n));

filename = 'frame'; % file name

for c = 1:n

imageStack(:,:,c) = rot90(double(imread([filename num2str(c-1) '.png'])));

c

end

**OpticalMapProcess2.m**

% Look at signals

figure;

imshow((imageStack(:,:,1)-min(min(imageStack(:,:,1))))./max(max(imageStack(:,:,1))));

axis equal

hold on;

frameperiod = 2.0; % camera frame period (ms)

% number of traces to show

ntraces = 7; % max of 7

tracecolor = ['b','r','g','c','m','y','k'];

roi = 12;

% select areas on heart tissue

for c = 1:ntraces

[x,y] = ginput(1);

row(c) = round(y);

col(c) = round(x);

plot([col(c), col(c)+roi-1], [row(c), row(c)], [tracecolor(c)]);

plot([col(c)+roi-1, col(c)+roi-1], [row(c), row(c)+roi-1], [tracecolor(c)]);

plot([col(c)+roi-1, col(c)], [row(c)+roi-1, row(c)+roi-1], [tracecolor(c)]);

plot([col(c), col(c)], [row(c)+roi-1, row(c)], [tracecolor(c)]);

end

for c = 1:ntraces

area = imageStack(row(c):row(c)+roi-1,col(c):col(c)+roi-1,:);

trace = mean(mean(area));

trace = reshape(trace,1,n);

T = 0:frameperiod:(n-1)*frameperiod;

figure;

plot(T, trace, [tracecolor(c)]); xlim([0 T(end)]);

end


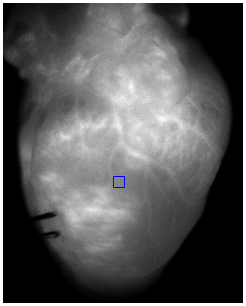

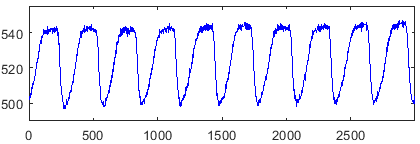


Sample voltage mapping data from a rabbit heart paced at a 300ms cycle length

**OpticalMapProcess3.m**

% Generate voltage or calcium movie

movieSequence = double(zeros(nrows,ncols,n));

for R = 1:nrows

for C = 1:ncols

trace = imageStack(R,C,:);

trace = reshape(trace,1,n);

% Normalize fluorescence signals

trace = max(trace) - trace; % voltage dye

%trace = trace - min(trace); % calcium dye

trace = trace/max(trace);

trace = smooth(trace,7,'moving'); % optional: signal smoothing

movieSequence(R,C,:) = trace;

end

R

end

**OpticalMapProcess4.m**

% Outline the region of interest around the heart

clear h_im

clear e

clear BW

clear testimage

testimage = imageStack(:,:,1);

figure

h_im = imshow((imageStack(:,:,1)-min(min(imageStack(:,:,1))))./max(max(imageStack(:,:,1))));

e = imfreehand(gca);

BW = createMask(e,h_im);

testimage = BW.*testimage;

figure; imshow((testimage-min(min(testimage)))./max(max(testimage)));

**OpticalMapProcess5.m**

% Show voltage or calcium movie

fh = figure;

imh = imshow(BW.*movieSequence(:,:,1), 'Colormap', hot);

truesize(fh,[300 300]);

for fr = 1:1:n

set(imh,'CData',BW.*movieSequence(:,:,fr)); drawnow();

title('Optical Mapping')

pause(0.04);

end


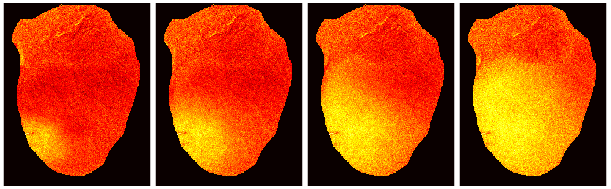


Some snapshots taken at 10ms intervals of the voltage mapping movie generated

**Hardware control software**

1. Start a new project in SimpleIDE using the provided C source code: OMSystem_v1.c

#include "simpletools.h"

#define DB0 0

#define DB7 7

#define RXF 8

#define RD 9

#define TXE 10

#define WR 11

#define SCK 12

#define SDI 13

#define CS1 14

#define CS2 15

#define LightSW 16

#define StimSW0 17

#define StimSW1 18

volatile T;

volatile int dT;

volatile int flag = 1;

volatile int stimState = 0; // electric stimulator state: OFF=0, ON=1

int *cogID;

// function prototypes

void EStimulate();

void DAC(int DAC_Select, int value, int clockLine, int dataLine, int selectLine);

int main()

{

char dataIn[5]; // byte array to hold data comming from PC

//-------------------------------------------------------------------------

set_directions(DB7,DB0,0b00000000); // input byte from UM245R

set_direction(RXF,0); // input, RXF

set_direction(RD,1); // output, RD

set_direction(TXE,0); // input, TXE

set_direction(WR,1); // output, WR

set_output(RD,1); // initialize RD

set_output(WR,0); // initialize WR

//-------------------------------------------------------------------------

set_direction(SCK,1); // output, SCK

set_direction(SDI,1); // output, SDI

set_direction(CS1,1); // output, chip-select 1

set_direction(CS2,1); // output, chip-select 2

set_direction(LightSW,1); // output, light switch

set_directions(StimSW1,StimSW0,0b11); // output, stimulator

set_output(SCK,0); // initialize SCK

set_output(SDI,0); // initialize SDI

set_output(CS1,1); // initialize chip-select 1

set_output(CS2,1); // initialize chip-select 2

set_output(LightSW,0); // initialize light switch

set_outputs(StimSW1,StimSW0,0b00); // intialize stimulator

//-------------------------------------------------------------------------

DAC(0,0,SCK,SDI,CS1); // initialize DAC to 0

DAC(1,0,SCK,SDI,CS1); // initialize DAC to 0

DAC(0,0,SCK,SDI,CS2); // initialize DAC to 0

DAC(1,0,SCK,SDI,CS2); // initialize DAC to 0

T = CLKFREQ; // default period of 1000 ms

dT = CLKFREQ/1000; // default pulse-width of 1 ms

unsigned int Tvalue = 0;

while(1)

{

for(int n=0; n<5; n++)

{

while(input(RXF)!=0){} // wait until data is received from PC

set_output(RD,0);

dataIn[n] = get_states(DB7,DB0); // read and store data byte

set_output(RD,1);

}

int DACvalue = 0;

switch(dataIn[0]) {

case 1:

DACvalue = dataIn[3] + dataIn[4]*256;

// Chip select: dataIn[1] = 0 ==> CS1

// dataIn[1] = 1 ==> CS2

DAC(dataIn[2],DACvalue,SCK,SDI,dataIn[1]+CS1);

break;

case 2:

set_output(LightSW,dataIn[1]);

break;

case 3:

Tvalue = dataIn[3] + dataIn[4]*256;

if (stimState==1)

{

flag = 0;

while(flag==0) {} // wait until it's OK to change T

T = CLKFREQ/1000*Tvalue;

}

else

T = CLKFREQ/1000*Tvalue;

break;

case 4:

Tvalue = dataIn[3] + dataIn[4]*256;

if (stimState==1)

{

flag = 0;

while(flag==0) {} // wait until it's OK to change dT

dT = CLKFREQ/1000*Tvalue;

}

else

dT = CLKFREQ/1000*Tvalue;

break;

case 5:

cogID = cog_run(EStimulate, 128);

stimState = 1;

break;

case 6:

if (stimState==1)

{

flag = 0;

while(flag==0) {} // wait until it's OK to shut down core

cog_end(cogID);

stimState = 0;

}

break;

default:

break;

}

}

}

// DAC4922 12-bit dual-channel digital-to-analog converter

void DAC(int DAC_Select, int value, int clockLine, int dataLine, int selectLine)

{

int mask = 0b100000000000;

set_output(selectLine,0); // select DAC chip

if (DAC_Select==0) // config bit 15, DACA or DACB select bit

set_output(dataLine,0);

else if (DAC_Select==1)

set_output(dataLine,1);

set_output(clockLine,1);

set_output(clockLine,0);

set_output(dataLine,0); // config bit 14, VREF unbuffered

set_output(clockLine,1);

set_output(clockLine,0);

set_output(dataLine,1); // config bit 13, 1x output gain

set_output(clockLine,1);

set_output(clockLine,0);

set_output(dataLine,1); // config bit 12, output enabled

set_output(clockLine,1);

set_output(clockLine,0);

for (int n=0; n<12; n++)

{

// send 12-bit data, MSB first

if(value&mask)

set_output(dataLine,1);

else

set_output(dataLine,0);

//set_output(dataLine,value&mask);

set_output(clockLine,1);

set_output(clockLine,0);

value <<= 1;

}

set_output(selectLine,1); // latch it in

}

void EStimulate()

{

set_directions(StimSW1,StimSW0,0b11); // output, stimulator

set_outputs(StimSW1,StimSW0,0b00); // intialize stimulator

int currentT = CNT + CLKFREQ/1000;

int tempT = currentT;

while(1)

{

waitcnt(currentT);

set_outputs(StimSW1,StimSW0,0b01);

tempT += dT;

waitcnt(tempT);

set_outputs(StimSW1,StimSW0,0b10);

tempT += dT;

waitcnt(tempT);

set_outputs(StimSW1,StimSW0,0b00);

currentT += T;

flag = 1;

tempT = currentT;

}

}

1. Set the *Project Options* and then upload the microcontroller program:


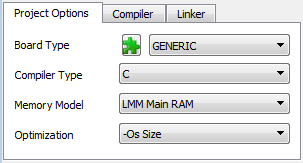

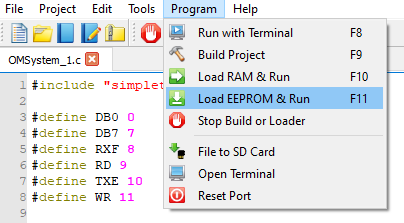


**MATLAB commands for communicating with your circuit board**

1. Construct a serial port object:

% Setting up COM port:

clear all;

s = serial('COM4'); % use the COM port assigned to your board by the OS

set(s,'BaudRate',115200);

% clear buffer

c = get(s,'BytesAvailable');

if c~=0

fread(s,c);

end

fopen(s) % to close the COM port, use fclose(s)

1. Set the brightness of the LEDs:

% Set light level

volt = 4.00; % set output voltage, use range: 0 - 7.49V

num = round((volt*4096)/(2.50*3)); % Vref = 2.5 V

% break up number into bytes

num2 = floor(num/256);

num1 = num - num2*256;

fwrite(s, [1 1 0 num1 num2], 'uint8'); % send command to controller board

1. Turn on the LEDs:

fwrite(s, [2 1 0 0 0], 'uint8'); % send command to turn ON

1. Turn off the LEDs:

fwrite(s, [2 0 0 0 0], 'uint8'); % send command to turn OFF

1. Set the flow-rate for peristaltic pump 1 (heart perfusion):

% Set peristaltic pump flow-rate

volt = 4.00; % set output voltage, use range: 0 - 9.99V

% setting the voltage to 0V will stop pumps

num = round((volt*4096)/(2.50*4)); % Vref = 2.5 V

% break up number into bytes

num2 = floor(num/256);

num1 = num - num2*256;

fwrite(s, [1 0 0 num1 num2], 'uint8'); % send command to pump 1

1. Set the flow-rate for peristaltic pump 2 (coronary effluent collection):

% Set peristaltic pump flow-rate

volt = 6.00; % set output voltage, use range: 0 - 9.99V

% setting the voltage to 0V will stop pumps

num = round((volt*4096)/(2.50*4)); % Vref = 2.5 V

% break up number into bytes

num2 = floor(num/256);

num1 = num - num2*256;

fwrite(s, [1 0 1 num1 num2], 'uint8'); % send command to pump 2

1. Set the electrical stimulation period (i.e. pacing cycle length):

num = 500; % set stimulation period in milliseconds

% break up number into bytes

num2 = floor(num/256);

num1 = num - num2*256;

fwrite(s, [3 0 0 num1 num2], 'uint8'); % send command to controller board


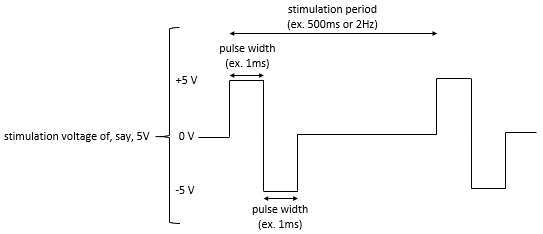


1. Set the biphasic stimulation pulse widths:

num = 1; % set width of upward and downward pulse, in milliseconds

% break up number into bytes

num2 = floor(num/256);

num1 = num - num2*256;

fwrite(s, [4 0 0 num1 num2], 'uint8'); % send command to controller board

1. Set the stimulation voltage:

% Set stimulation voltage

volt = 5.00; % set output voltage, use range: 0 - 19.99V

num = round((volt*4096)/(2.50*8)); % Vref = 2.5 V

% break up number into bytes

num2 = floor(num/256);

num1 = num - num2*256;

fwrite(s, [1 1 1 num1 num2], 'uint8'); % send command to controller board

1. Turn on electrical stimulation:

fwrite(s, [5 0 0 0 0], 'uint8'); % send command to turn ON stimulator

1. Turn off electrical stimulation:

fwrite(s, [6 0 0 0 0], 'uint8'); % send command to turn OFF stimulator

**Web links for sample pig and rabbit movies:**

Rabbit Voltage Pacing

https://figshare.com/s/0bc75c905bf74cf051e1

DOI (once it is made public): 10.6084/m9.figshare.14977896

Rabbit Calcium Pacing

https://figshare.com/s/739b17c659a92d108313

DOI (once it is made public): 10.6084/m9.figshare.14977881

Rabbit Calcium VF

https://figshare.com/s/aa1970a96af537a428ea

DOI (once it is made public): 10.6084/m9.figshare.14977893

Pig Voltage Pacing

https://figshare.com/s/672a2ab3340bf95b612c

DOI (once it is made public): 10.6084/m9.figshare.14977875

Pig Voltage VF

https://figshare.com/s/178a8b1cddcf0c39cc13

DOI (once it is made public): 10.6084/m9.figshare.14977878

**Legends for the supplemental movies:**

**Suppl. Movie S1:** Pig heart voltage optical mapping during paced (left panel) and fibrillatory (right panel) activity.

**Suppl. Movie S2:** Rabbit heart voltage optical mapping during paced (left panel) and fibrillatory (right panel) activity.

**Suppl. Movie S3:** Rabbit heart calcium optical mapping during paced (left panel) and fibrillatory (right panel) activity.
